# Innovative approach: utilizing silver nanoparticles sheet for improved rabbit cecal anastomosis healing

**DOI:** 10.3389/fvets.2024.1264414

**Published:** 2024-02-26

**Authors:** Zakriya Almohamad, Reham Fahmy, Amany Farag, Ahmed Abdellatif, Wael Mossallem, Abdelnaser A. Abdallah, Marwa Abass

**Affiliations:** ^1^Department of Clinical Sciences, College of Veterinary Medicine, King Faisal University, Al Ahsa, Saudi Arabia; ^2^Veterinary Surgery, Oncology Centre, Mansoura University, Mansoura, Egypt; ^3^Department of Cytology and Histology, Faculty of Veterinary Medicine, Mansoura University, Mansoura, Egypt; ^4^Department of Anatomy and Embryology, Faculty of Veterinary Medicine, Mansoura University, Mansoura, Egypt; ^5^Veterinary Clinical Supervisor, Al-Raha Veterinary Clinic, Abu Dhabi, United Arab Emirates; ^6^Department of Internal Medicine and Infectious Disease, Veterinary Teaching Hospital, Faculty of Veterinary Medicine, Mansoura University, Mansoura, Egypt; ^7^Department of Surgery, Anesthesiology, and Radiology, Faculty of Veterinary Medicine, Mansoura University, Mansoura, Egypt

**Keywords:** rabbit model, cecum, transection and anastomosis, healing, silver nanoparticles sheet

## Abstract

**Introduction:**

Anastomotic leakage is a severe complication associated with gastrointestinal surgery. The process of intestinal wound healing is crucial for the successful outcome of digestive tract surgical repair procedures. This research aimed to determine the impact of silver nanoparticles sheet (Acticoat) on the anastomotic healing of the cecum in rabbits.

**Methods:**

A total of 48 New Zealand male rabbits in good health were used for cecum transection and anastomosis. The animals were randomized into the control group (C) and the silver nanoparticles group (AgNPs). In the C group, the transected cecum was end-to-end anastomosed with a single layer of simple continuous suture pattern using 3–0 polyglyconate. In contrast, a silver nanoparticle sheet (Acticoat) was covered around the sutured anastomotic line in the AgNPs group. Postoperatively, abdominal ultrasound imaging and the Bristol Rabbit Pain Score (BRPS) were measured on days 7, 15, and 30. Eight rabbits from each group were euthanized at each time point to assess macroscopic findings, bursting pressure tests, tensile strength tests, histopathological examinations, and immunohistochemical analyses.

**Results:**

The AgNPs group demonstrated a significant increase in the cecal lumen diameter wall (*p* ≤ 0.001), burst pressure measurement (*p* ≤ 0.02), and tensile strength (*p* ≤ 0.01). Conversely, the AgNPs group had significantly lower BRPS scores (*p* ≤ 0.01). In addition, histopathological examinations revealed that AgNPs significantly reduced inflammatory cell infiltration (neutrophils and macrophages) and enhanced collagen deposition. Immunohistochemical analyses revealed a significant increase (*p* ≤ 0.01) of α-SMA and a reduction of CD31 in the anastomotic tissue of the AgNPs group.

**Discussion:**

The results of the present study indicate that the utilization of the AgNPs sheet (Acticoat^®^) effectively enhanced the strength of cecum anastomosis, resulting in a reduction in anastomosis leakages, pain scores, and abdominal adhesions. Additionally, the bursting pressure values in the rabbit model were significantly increased.

## 1 Introduction

Acute gastrointestinal obstruction or gastrointestinal disorders, commonly called gastrointestinal dilation or bloat, is a critical, life-threatening condition in pet rabbits, primarily located in the duodenum, ileocecal-colonic junction, cecum, or colon. Complete cecal obstipation is a life-threatening condition that necessitates immediate intervention (1). Therefore, surgical removal is often necessary in unresponsive cases. However, anastomotic leakage remains one of the most significant issues in gastrointestinal surgeries (2). The primary reasons for anastomotic dehiscence are surgical techniques, local oxygen concentration, bacterial contamination, and associated diseases of the operated patients. Preventing anastomotic leaking is more crucial than treating anastomotic dehiscence because it reduces the need for repeated laparotomies and raises the risk of morbidity and mortality (2, 3).

In addition, intra-abdominal adhesions are frequently asymptomatic and considered a common cause of chronic abdominal pain and bowel obstruction, making postoperative abdominal adhesion one of the most significant causes of concern (4, 5). The tissue healing process is defined by tissue remodeling, granulation formation and proliferation, inflammation, and coagulation. Inflammation is a crucial factor in all cases. However, persistent inflammatory responses may postpone wound healing due to the infiltration of inflammatory cells and the upregulation of proinflammatory cytokines (6).

A recent study focused on accelerating wound healing using nanomaterials such as zinc (Zn), silver (Ag), and copper (Cu). AgNPs have been widely used in medical dressings to treat wounds (7). Consequently, some anti-inflammatory agents, including AgNPs, have been utilized to accelerate healing (8–14). Previous research has proven that AgNPs-coated sutures had an enhanced anti-inflammatory impact on intestinal anastomosis, particularly in the early healing phase (11). Numerous silver-enhanced dressings with antibacterial characteristics involving alginate-based materials have been introduced to the pharmaceutical market (14).

Nevertheless, the majority of commercially available items contain silver in an ionic form, and there are a limited number of dressings containing nanocrystalline silver, such as the collagen-based Acticoat^®^ [Smith & Nephew-London-United Kingdom; (15)], which has proven to be effective in wound management. Furthermore, AgNPs-coated sutures may provide an optimal environment for intestinal anastomosis healing in mice (16).

Alginates are linear polysaccharides produced by Phaeophyceae (brown marine algae) and are widely used as stabilizing and thickening agents in the chemical and food industries. Recently, alginate has been found to have an excellent anti-adhesive effect and has been used in a rat model of uncompromised anastomotic colon healing with no interference with the anastomotic repair (17–19). In addition to its safe use in visceral surgery, studies have shown that alginate does not interfere with anastomotic healing under various conditions (20). Acticoat^®^ (Smith and Nephew United States, Largo, FL) is an alginate dressing impregnated with nanocrystalline silver crystals designed to provide a controlled silver release with a well-known bactericidal effect. The Acticoat^®^ is applied wet and left in place. It is saturated multiple times per day to enable the release of silver, which has an antimicrobial impact lasting for 7 days. Contrary to the manufacturer’s recommendations, previous studies have left the Acticoat^®^ in place as long as it remained adherent (21–23).

There were no adverse effects associated with this practice, and it is predicted that patients treated with Acticoat^®^ would experience less pain during dressing changes than those treated with other dressings without adversely affecting wound healing (14). The main goal of many previous approaches (24, 25) as well as the present study was to alleviate complications such as adhesion occurrence, severity, and extent by preventing contamination and maintaining the normal healing process. To the best of the authors’ knowledge, no previous studies have evaluated the effect of Acticoat^®^ on intestinal healing yet. Thus, the current research focuses on studying the impacts of Acticoat^®^ sheet on cecal anastomosis in rabbits.

## 2 Materials and methods

**Figure 1:**
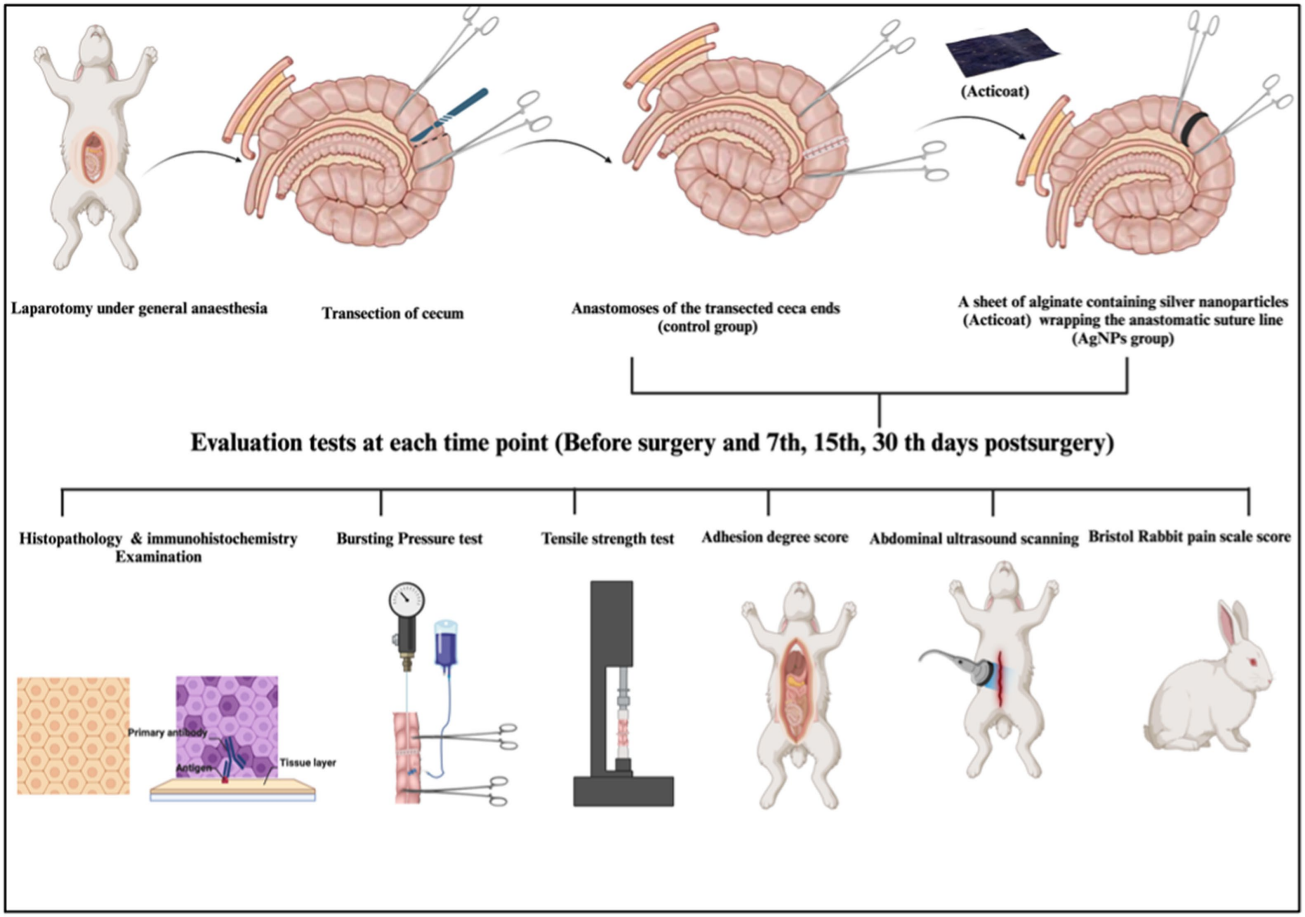
The schematic cartoon of the experimental strategy. The author of the manuscript designed it. (Created with Biorender.com with permission).

All experiments were performed following relevant guidelines and regulations. The Welfare and Ethics Committee approved this study, which was reviewed and approved by the Mansoura University Animal Care and Use Committee with a documented code MU-ACUC (MV.R.20.03.110). All procedures were reported following the format specified by ARRIVE (26). The schematic cartoon of the experimental strategy is represented in Figure 1.

### 2.1 Animals

**Figure 2:**
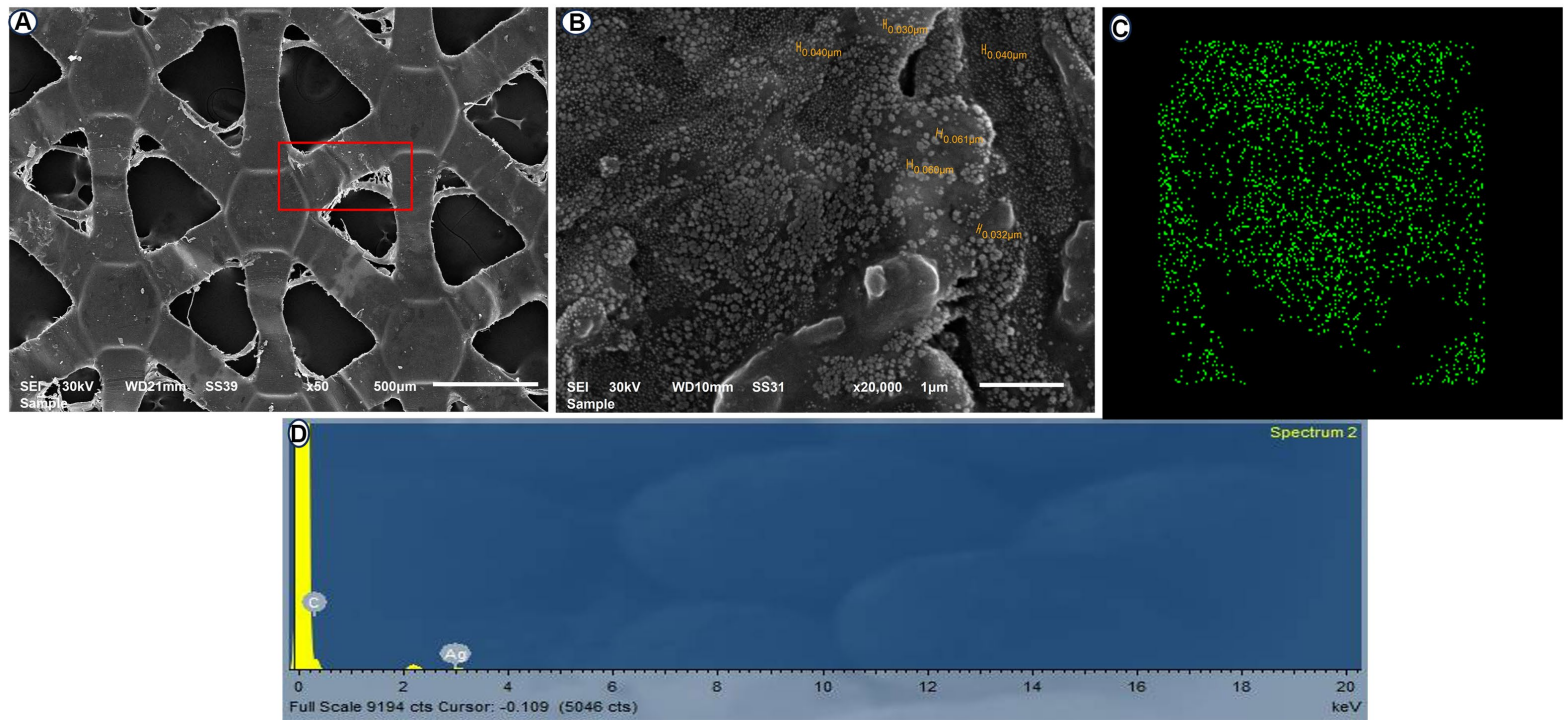
Scanning electron microscopy (SEM) of the ACTICOAT sheet detects the morphology of AgNPs at 50X **(A)** and 20,000X **(B)**. EDX mapping of addressed silver nanoparticles **(C)**. Energy dispersive X-ray spectroscopy (EDX) spectrum analysis showing silver nanoparticles **(D)**.

Forty-eight male healthy New Zealand rabbits (3.9 ± 0.2 kg and 8 ± 3 months old) were used in this study. The rabbits were randomly assigned to two groups (*n* = 24/group). After transection of the healthy cecum, the cecum was end-to-end anastomosed with a single layer of a simple continuous suture pattern using 0–3 polydioxanone suture material (PDS; *p*-Dioxanone; EGY-PDS, Egypt) in the control group (C). In the silver nanoparticles (AgNPs) group, a sheet of alginate containing silver nanoparticles (Acticoat, Absorbent with SILCRYST, Smith &Nephew Ltd., London, United Kingdom; Figure 2) was covered over the suture anastomotic line.

### 2.2 Properties of Acticoat (nanocrystalline silver)

Acticoat is prepared using the physical vapor deposition method. An anode is created by introducing argon gas into a vacuum chamber. By introducing an electric current into the chamber, the argon ions release the silver atoms that move toward the surface to be covered. This process results in the deposition and formation of nanocrystals, each with a diameter of 15 nm, consisting of approximately 30 to 50 atoms. The alterations made to the lattice structure of the crystal led to the formation of a highly energetic and meta-stable state of elemental silver. When Acticoat is wet and applied to the wound, it produces clusters of highly reactive silver cations at a concentration of up to 100 parts per million. This leads to electron transport, which, in turn, results in the inactivation of bacterial cell DNA, damage to the cell membrane, and the formation of insoluble complexes that bind to microorganisms. Compared to other types of silver, such as 0.5% silver nitrate or silver sulfadiazine, Acticoat releases 30 times fewer silver cations. This leads to a longer-lasting and more effective release of silver. When Acticoat is re-moistened, the clusters of silver release cations onto the wound for up to 7 days (27, 28).

### 2.3 Surgical procedures

All experimental surgeries began at the same time in the morning to minimize the impact of circadian rhythm. Before each experiment, rabbits were fasted for 12 h prior to surgery and up to 24 h after surgery. Before surgery, all rabbits were given an intramuscular injection of 20 mg/kg of amoxicillin (Betamox, Norbrook Company, United Kingdom). During surgery, 10 mg/kg of metronidazole (Flagyl, Aventis) was administered intravenously.

**Figure 3:**
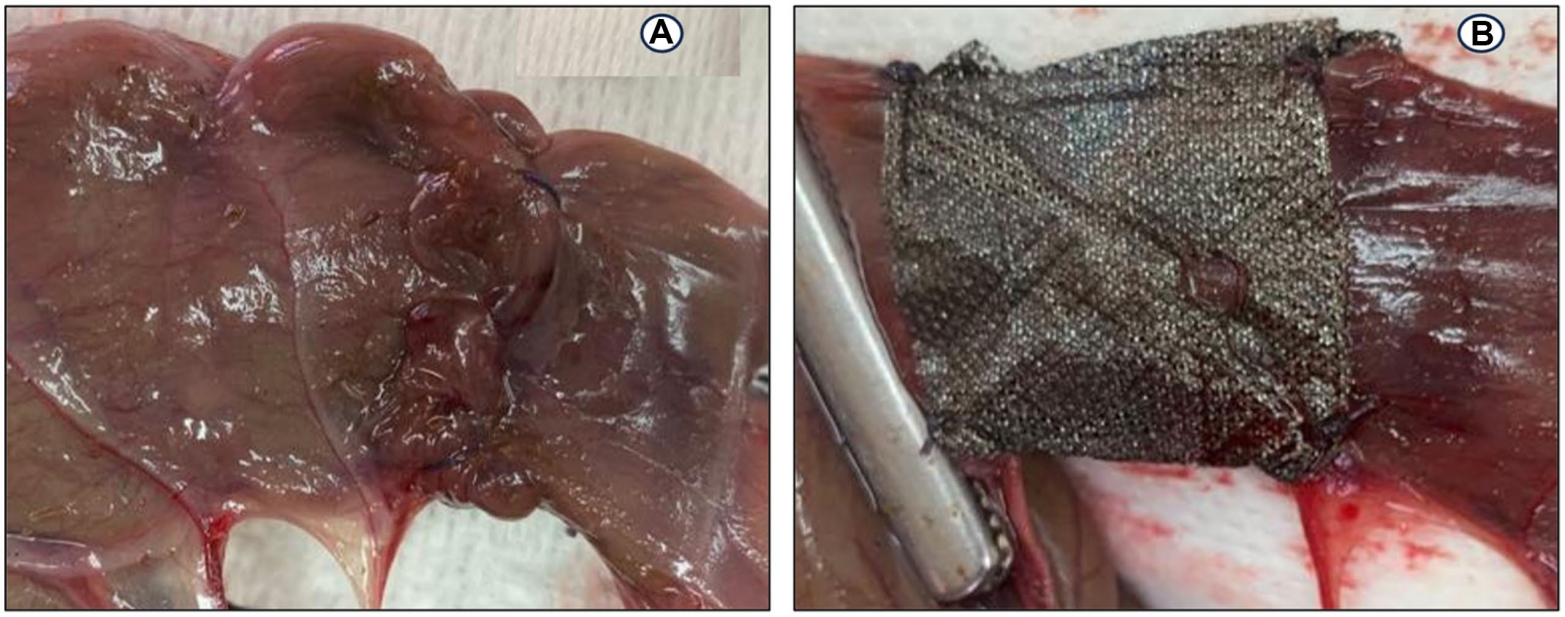
End-to-end anastomosis on the cecum in a rabbit model: **(A)** Figure-of-eight anastomosis; **(B)** a silver nanoparticles sheet (Acticoat) wrapping on the anastomotic suture line.

The rabbits were sedated with an intramuscular injection of 0.02 mg/kg acepromazine (Castran 1.5%; Interchemie Co., Holland) and 0.2 mg/kg methadone (Physeptone injection, methadone 1%, Martindale Laboratories, United Kingdom); 15 min later, a 24-gauge catheter (Jelco, Smith Medical International Ltd., United Kingdom) was introduced into a marginal ear vein. Three mL/kg/h of lactated Ringer’s solution (Ringer’s Injection, 500 mL, Al Mottahedoon Pharma, Egypt) was infused through the venous access until complete recovery. A combination of midazolam (1 mg/kg; Midathetic 5%, Amoun Company, Egypt), xylazine (10 mg/kg; Xylaject 2%; Adwia Company, Egypt), and ketamine (75 mg/kg; Narketan 10%, Chassot AG, Belp-Bern, Switzerland) was injected intramuscularly to induce anesthesia in the rabbits. An aseptic surgical preparation of the rabbits’ abdomens was performed. A 5-cm midline abdominal laparotomy was created, and all the abdominal viscera were carefully examined. At the right flank of the abdominal cavity and caudally near the pelvic cavity, the second segment of the cecum was detected and pulled out from the surgical incision. Before the cecum transection, its contents were gently pressed toward the distal part of the cecum. Two intestinal forceps were used to grasp the intended cecum part, spaced apart by 5 cm. A full-thickness transection was made in a mesenteric-to-antimesenteric orientation. Wet swabs were used to remove the ingesta, and the everting mucosa was trimmed. The two ends of the transected cecum were anastomosed with a single-layer figure-of-eight pattern (Figure 3A) using a 3–0 polydioxanone absorbable suture material as described by Liu et al. (10).

In the AgNPs group, a 2 × 5 cm piece of Acticoat sheet was placed in the anastomotic suture line (Figure 3B). No fortifying material was used in the C group. The cecum was carefully repositioned into the abdominal cavity, and wet swabs were used to manipulate the viscera throughout the procedure.

Abdominal layers and skin wounds were sutured routinely. Rabbits recovering after surgery were kept in separate cages and, after an overnight fast, had access to food and water. All rabbits were intravenously injected with amoxicillin (20 mg/kg; Augmentin; 1.2 g; GlaxoSmithKline; Ireland), cefazolin (22 mg/kg; Zinol; 1 g; Pharco B International; Egypt), and meloxicam (0.2 mg/kg; Metacam; 5 mg/mL; MSD CO; France) every 12 h for 5 days. A trained, blinded veterinarian monitored the rabbits’ daily physical activities, vital signs, and abdominal wounds.

### 2.4 Ultrasound scanning

Rabbits were placed in dorsal recumbency for each ultrasonographic examination, their hair was clipped, and acoustic sterile coupling gel was applied. The rabbits were manually restrained for the duration of the ultrasonographic examination. All ultrasound examinations were performed using an 8–13 MHz linear transducer (MINDRAY DP-2200Vet, China). Sonographic tests were performed before surgery and postoperatively on the 7th, 15th, and 30th days. Cecum diameter (mm) at the line of anastomosis was measured by placing the calipers of the ultrasound machine software on the serosal and mucosal surfaces perpendicular to the bowel wall axis. A complete abdominal ultrasonographic examination was performed with the animal in both right and left lateral recumbency. The stenotic degree was scored as described in Table 1.

**Table 1:** Stenotic degree and score of the anastomotic cecum.

Stenotic degree	Cecum diameter	Score
Normal	≥ 30 mm	0
Mild	20:30 mm	1
Moderate	10:20 mm	2
Severe	≤ 10 mm	3

### 2.5 Postoperative pain scale and clinical assessment

Based on Benato et al. (29) studies, rabbits were subjected to clinical evaluation on the day before surgery, the 7th, 15th, and 30th days post-operation. The Bristol Rabbit Pain Score (BRPS) was used as a composite pain measure that included 6 categories (behavior, grooming, ears, posture, eyes, and locomotion) and 4 pain score intensities (0, 1, 2, and 3) for a total score of 0 to 18.

### 2.6 Necropsy gross findings

Rabbits (*n* = 8/group) were euthanized by IV injection of 200 mg/kg thiopental Na (Thiopental, 0.5 gm vial, EIPICO Pharmaceutical Industrial Company, Egypt) at the 7th, 15th, and 30th day postoperatively (30). During necropsy, the cecal anastomotic region was examined objectively in a blinded fashion depending on the modified adhesion scale (31). The scale, graded from 0 to IV, measured the severity of anastomotic adhesions, stenosis, dehiscence, and intestinal obstructions. Grade 0 referred to no adhesion, Grade I referred to adhesion occurring between an anastomotic line and one abdominal organ, Grade II referred to adhesion occurring between an anastomotic line and two abdominal organs, Grade III referred to adhesion occurring between an anastomotic line and more than two abdominal organs, and Grade IV referred to multiple intra-adhesions between the abdominal organs and anastomotic line. The average total score in each group was calculated, and then a mean adhesion score for each group was determined.

### 2.7 Anastomotic bursting pressure

A 15-cm section before and after the cecum anastomotic line was separated, and its contents were evacuated and filled with normal saline. One end of the separated cecum was connected to a pressure gauge and the other to an infusion pump that injected 8 mL/min of methylene blue. The anastomotic rupture pressure was recorded as the bursting force of the dye solution from the anastomotic line (32).

### 2.8 Anastomotic site tensile strength

The 3 cm before and after the cecum anastomotic line was fixed to a tensile test machine (an AG-X Plus Desktop 10 kN, Pneumatic Grip, Shimadzu, Japan) at a rate of 60 mm/min until rupture occurred. The weight necessary to break the anastomotic tissue was recorded as the tissue tensile strength (33).

### 2.9 Histopathological examination

After euthanizing the animals, intestinal biopsies were taken to assess cecum morphology. A 3-cm segment of the cecum, including the anastomotic site, was extracted from the experimental groups (C and AgNPs groups) on the 7th, 15th, and 30th days following surgery. These specimens were fixed for 24 h in a 10% neutrally buffered formalin fixative and then embedded in paraffin. The proliferative and healing phases of the anastomotic wound were evaluated by staining paraffinized sections with hematoxylin and eosin (H&E), followed by trichrome stains to highlight the standard tissue architecture and collagen fiber deposition, according to Bancroft and Gamble (34).

Other sections were immunohistochemically stained with two antibodies: CD31 (1:100; Thermo Scientific) to determine the number of microvessels and monoclonality and smooth muscle actin α-SMA (1:100; Boster Immunoleader) to determine the presence of α-SMA in anastomotic tissue. Under a microscope, histological examinations of all stained sections were performed using an Olympus CX 41. ImageJ version 1.36 (NIH, United States) was used to analyze quantitative immunohistochemicals. For this evaluation, five fields per five stained sections for each group were analyzed based on the Allred score employed by Fedchenko and Reifenrath (35). ImageJ (NIH, United States) and Microsoft Excel 2016 software (United States) were utilized to evaluate all morphological data statistically.

### 2.10 Statistical analysis

The data were analyzed using IBM SPSS software package version 28.0, IBM Corp. in Armonk, NY. Numerical variables were tested for normalcy using the Shapiro–Wilk test. Numbers and percentages were used to describe qualitative data, whereas the mean, standard deviation, and standard error were presented for quantitative data. The relationship between two categorical variables was examined using the chi-square test. The continuous variables within the different measurements of variables and between the study groups were compared by using a repeated-measures ANOVA. Pairwise comparisons between the statistically significant measurements were performed using the Bonferroni test, with values ≤0.05 being regarded as statistically significant.

All statistically significant changes between the two groups were compared using an independent-sample *t*-test. Statistical significance was defined as *p*-values ≤0.05. Standard error ± mean was used to express the data.

## 3 Results

### 3.1 Ultrasound scanning

**Figure 4:**
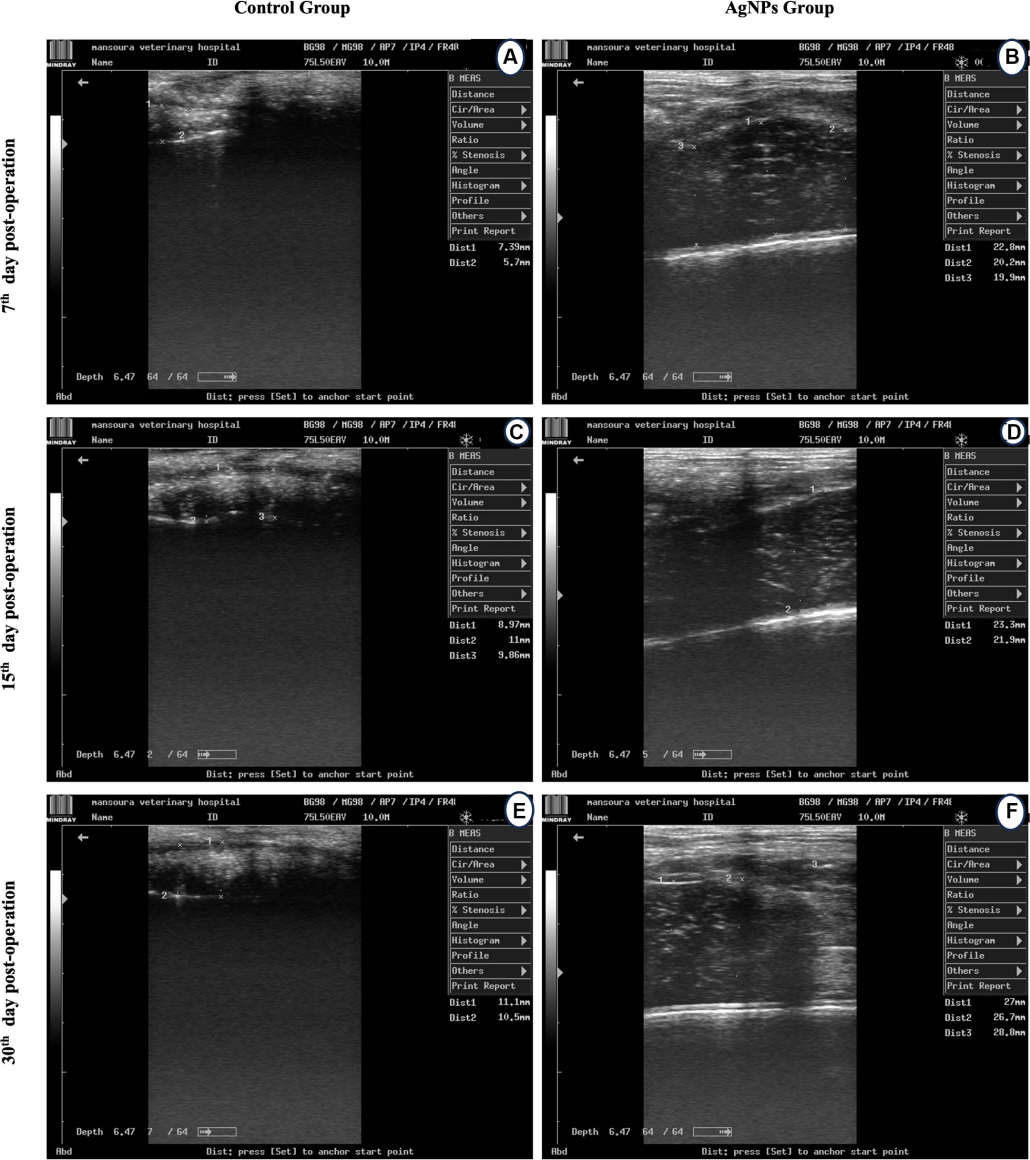
Ultrasound scanning for measuring cecum lumen diameter at the anatomic line in the control **(A,C,D)** and the AgNPs **(B,E,F)** groups at 7-, 15-, and 30-days post-operative.

The cecum diameter before surgery in rabbits was 30.6 ± 0.23 mm. Cecum transection and anastomosis caused a significant decline (*p* ≤ 0.003) in the cecum diameter in both groups. Moreover, the cecum diameter at the anastomotic line in the C group was significantly decreased (*p* ≤ 0.001) compared to the AgNPs group. The diameter of the cecum anastomotic line in the C group was 6.5 ± 0.23, 9.1 ± 0.15, and 10.8 ± 0.3 mm versus (20.9 ± 0.6, 22.6 ± 0.41, and 27.5 ± 0.82 mm) in the AgNPs group, respectively, which were recorded at 7th, 15th, and 30th days post-surgery (Figure 4). The strontic score was 1 in the AgNPs group while that in the control group was 2–3.

### 3.2 Postoperative pain scale and clinical assessment

**Figure 5:**
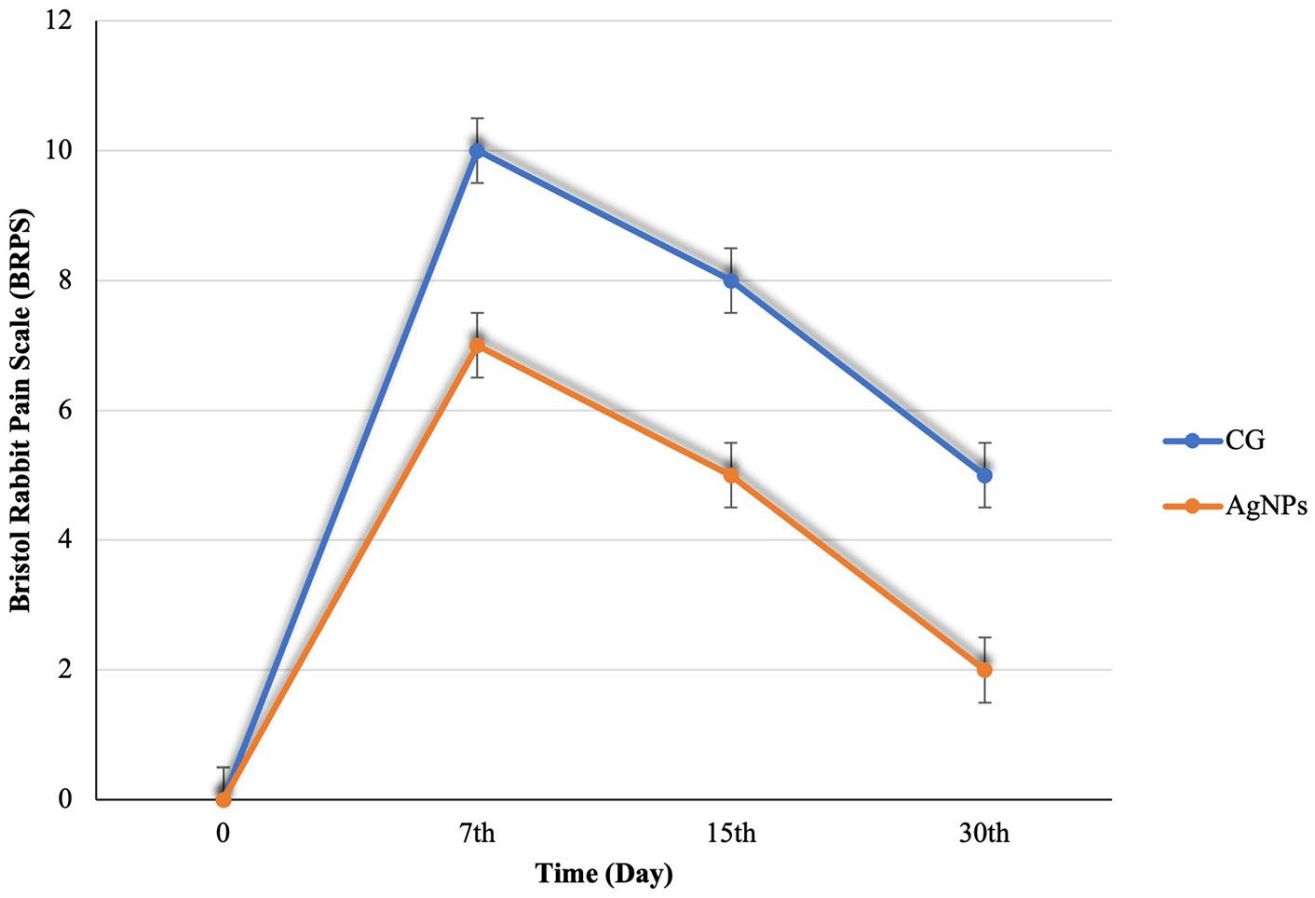
Bristol Rabbit Pain scale (BRPS) scores in the control and AgNPs groups at 7-, 15-, and 30-days post-operative.

No serious postoperative complications were noted during the 30th day post-surgery. BRPS scores were recorded, and the average scoring time ranged between 4 and 10 min, averaging 5 min. Prior to surgery, there was no variation in the pain scale scores of the rabbits. Postoperatively, the BRPS scores in the AgNPs group were considerably lower (*p* < 0.01) than in the C groups (Figure 5). Furthermore, the orbital tightening that occurred when the eye was closed was more prominent and the ears were curved and may be facing either side or back of the body in the C group. On the 7th day following surgery, the rabbits’ dullness and lethargy, resistance to movement, and hunched postures were also noted in the C group.

### 3.3 Necropsy gross findings

**Figure 6:**
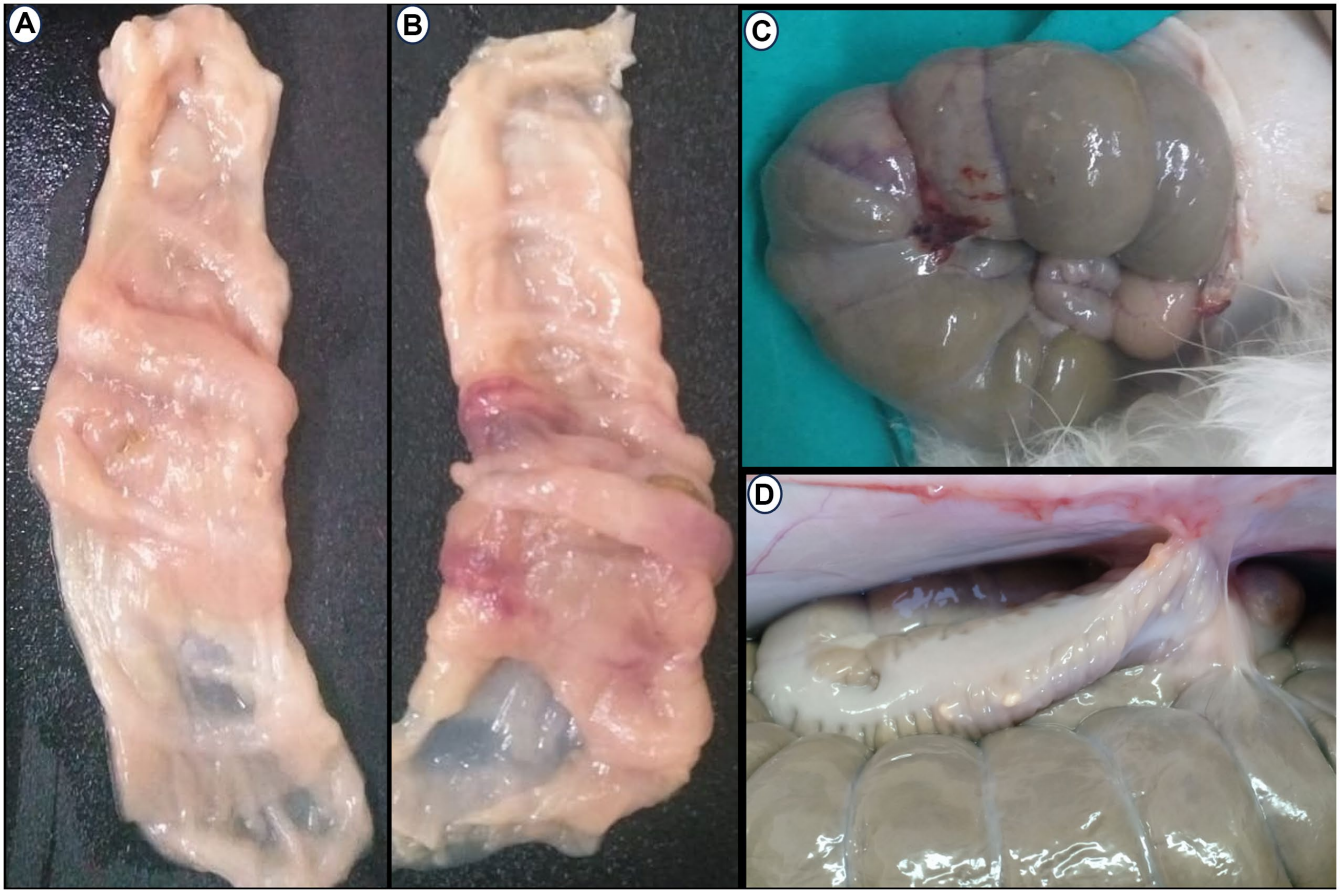
Postoperative Macroscopic findings: **(A–C)** Thickness and hemorrhagic area appear in the cecum wall at anastomotic line in the control group. **(D)** Adhesion between the cecal anatomic line, abdominal wall, and colon.

Postoperatively, anastomotic cecal wall thickness and hemorrhage were manifested in the C group (Figures 6A–C). None of the rabbits in the AgNPs group showed any anastomotic leakage (100%), whereas only five rabbits (20.8%) in the C group had no signs of anastomotic leakage. The adhesion intensity was significantly higher (*p* ≤ 0.001) in the C group than in the AgNPs group at each measuring point. Two rabbits (score = 0; 8.3%) in the C group had no signs of adhesions; four rabbits (score = 1; 16.7%) had adhesions between the cecum anastomotic line and the abdominal wall, whereas six rabbits (score = 2; 25%) showed adhesions between the anastomotic line, liver, and the abdominal wall. The cecum anastomotic line was entirely attached to the colon, small intestines, and abdominal wall in the remaining 12 rabbits (score = 3; 50%). In contrast, in the AgNPs group, the cecum anastomotic line was mobile and free of adhesion (score = 0) in 17 rabbits (70.9%), while adhesion was present in 4 rabbits (score = 1; 16.6%). The other three rabbits had adhesion (score = 2; 12.5%) between the anastomotic line and colon (Figure 6D).

### 3.4 Bursting pressure of anastomotic part (BP)

**Figure 7:**
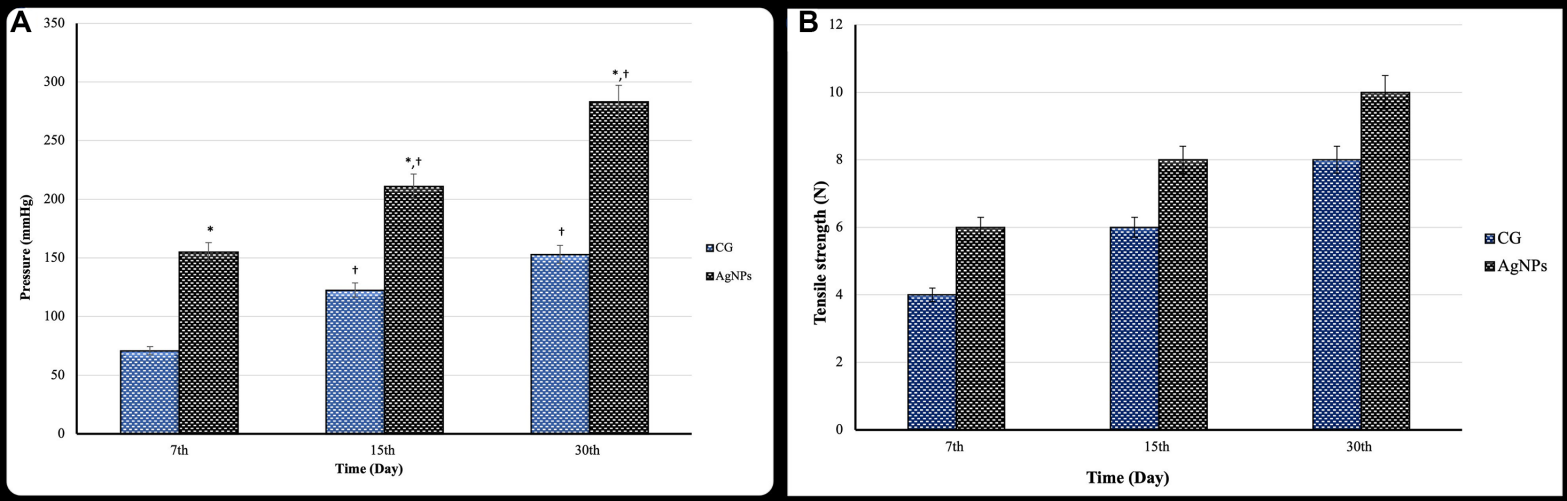
**(A)** Bursting pressure (mmHg) and **(B)** tensile strength test (*N*) of the cecal anastomotic line in the control and AgNPs groups at 7-, 15-, and 30-days post-operative (mean ± SD).

Bursting pressure was significantly elevated (*p* ≤ 0.05) within the same group as the measurement time points increased. The anastomotic sites significantly increased (*p* ≤ 0.02) the bursting pressure in the AgNPs group compared to the C group on the 7th, 15th, and 30th days postoperatively (Figure 7A).

### 3.5 Anastomotic tensile strength

The anastomotic tensile strength increased significantly (*p* ≤ 0.05) in both groups over time post-operation. Furthermore, there was a considerably higher anastomotic tensile strength in the AgNPs group (*p* ≤ 0.01) than in the C group during the trial on the 7th, 15th, and 30th days postoperatively (Figure 7B).

### 3.6 Microscopic findings

The morphological changes at the anastomotic sites were meticulously followed up over the 30 days postoperatively. Both groups demonstrated discernible healing; however, the AgNPs group showed a recognizable improvement, characterized by the synchronous union of leukocytic cells, fibroblasts, and endothelial cells of blood-forming tissue, reducing the inflammatory response.

**Figure 8:**
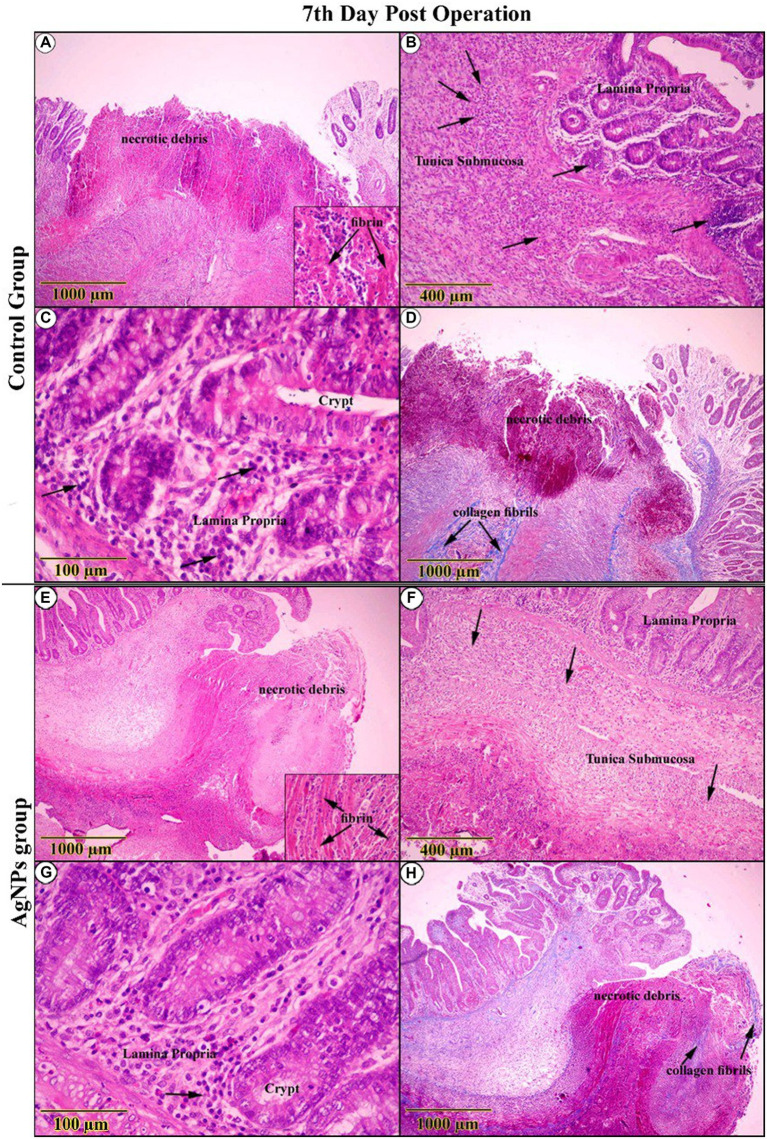
H&E-stained sections on the 7th day post-operation, in the control group showed **(A)** necrotic debris was confined within an eosinophilic fibrin network (Inset). **(B,C)** The complete cecal tunic was severely inflamed (Arrows). Around the anastomotic margins, the trichrome-stained section revealed faintly blue collagen fibrils (Arrows) **(D)**. On the other side, in the AgNPs group revealed **(E)** bundles of fibrin dominated at the expense of diminishing necrotic debris (Inset). **(F,G)** The complete cecal tunic was mildly inflamed (Arrows). Around the anastomotic margins, the trichrome-stained section revealed faintly blue collagen fibrils invading the necrotic tissue (Arrows) **(H)**.

The gross examination of the anastomotic site on the 7th day in the C group revealed severely congested blood vessels. Microscopic examination of the wound gap showed necrotic debris composed of neutrophils admixed with many erythrocytes, trapped in the exudate of thick, strand-like material of eosinophilic fibrinous mesh (Figure 8A). Acute cecal inflammation was visible around the wound margins, with more neutrophils, fibroblasts, histiocytes, plasma cells, and eosinophils within the epithelium and lamina propria (Figure 8B). Mild architectural deformation and apoptosis were observed in the crypts, with no evidence of goblet cells in either the crypt epithelium or the surface epithelium (Figure 8C). The mucosal–submucosal interface was rich in lymphoid follicles, and a faint blue collagen fibril could be seen close to the anastomotic line, though these fibrils did not appear in the wound gap (Figure 8D). The wound gap in the AgNPs-treated group showed conspicuous bundles of fibrin threads on the 7th postoperative day, accompanied by a decrease in necrotic debris (Figure 8E). The entire tunic exhibited less damage from inflammation at the incision borders (Figure 8F).

Conversely, there was a striking rise in epithelial thickness due to modest cellular hyperplasia and various cellular mitoses. Several cellular components deviated from standard shape features (reactive atypia; Figure 8G). Collagen fibers were found to have significantly increased in number close to the anastomotic line and extended into the necrotic tissues that spanned the wound gap (Figure 8H).

**Figure 9:**
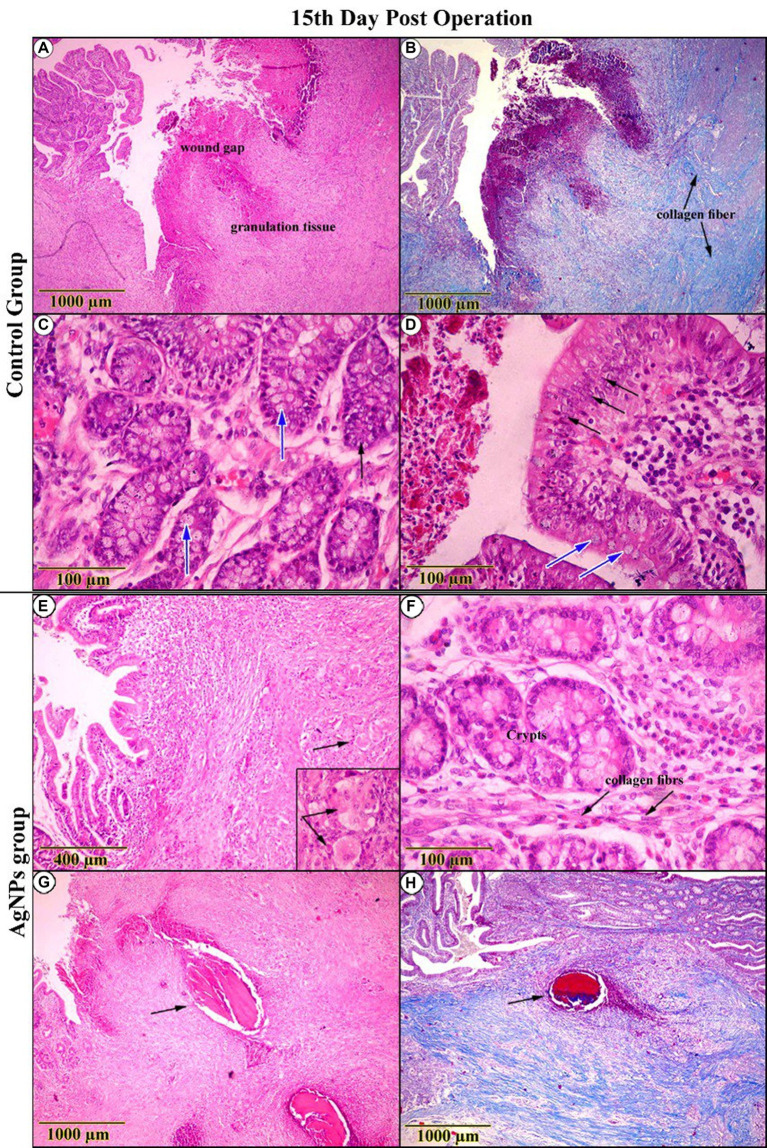
Histopathological examination (H&E and trichrome-stained) on the 15th day post-operation. In the control group, appeared **(A)** a conspicuous retraction of the wound gap and an increase in the proliferation of granulation tissue. **(B)** Trichrome-stained sections revealed an increment in collagen fibers **(C,D)**. High-magnification sections showed cellular hyperplasia (Arrow) and a notable number of goblet cells (Blue-Arrow). Conversely, in the AgNPs group, it seemed **(E,F)** a high proliferation rate of granulation tissue, as indicated by the presence of histiocytes (Inset). **(G,H)** As a result of suture material resorption, sections revealed the presence of solitary vacuoles surrounded by leukocytic infiltration (Arrow).

On the 15th day post-operation, the C group examination displayed conspicuous retraction of the wound gap area due to increased proliferative granulation tissue. Both the surface epithelium and crypts were crowded with cellular hyperplasia, accompanied by notable numbers of goblet cells (Figures 9A,C,E,G). In the AgNPs-treated group, examination revealed an outstanding exaggeration of proliferation and regeneration rates among the entire intestinal wall, confirmed by the presence of histiocytes. As a result of the resorption of suture material, the tunica mucosa contained many vacuoles surrounded by solitary areas of leukocytic infiltration (Figures 9B,D,F,H).

**Figure 10:**
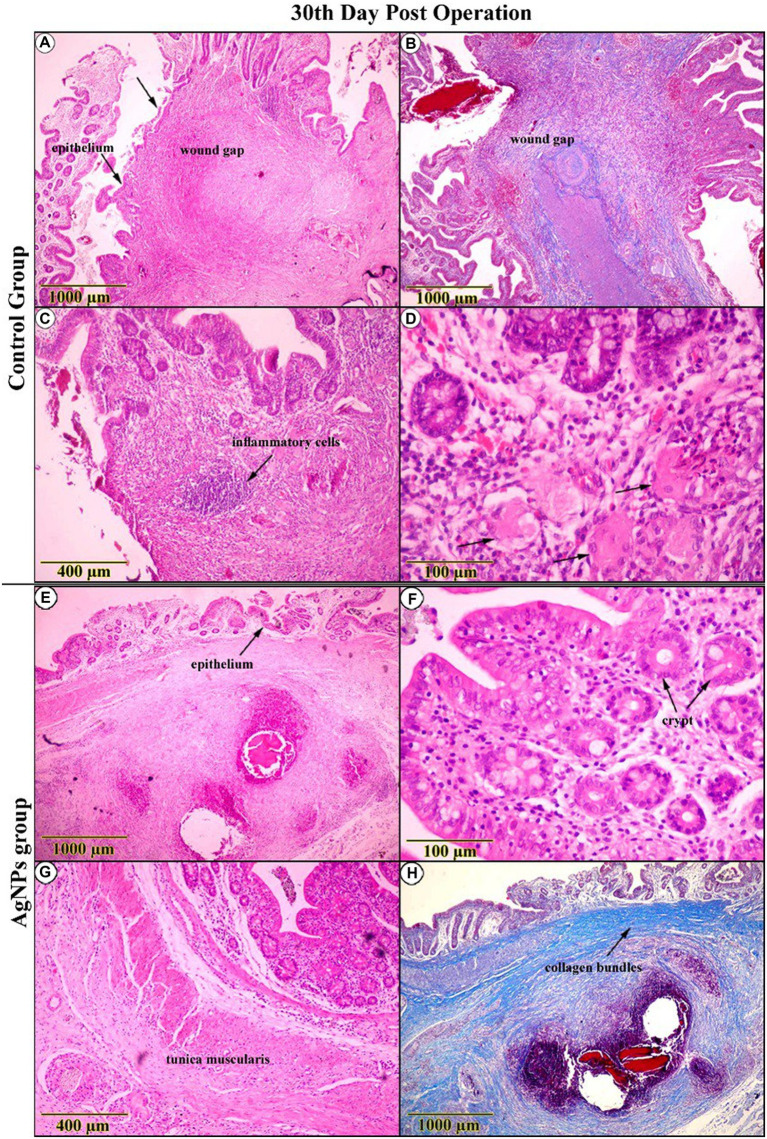
Histopathological examination (H&E and trichrome-stained) on the 30th-day post-operation in the control group **(A–D)** indicated that the cecal wall had improved, with surface epithelial repair over the wound gap, indicating overall improvement despite the presence of inflammatory signs and histiocytes (Arrows). **(E–H)** While in the AgNPs group, the entire wall appeared completely reconstructed. The superficial and crypt epithelium resembles the typical architecture with interconnected collagen bundles. Tunica muscularis arrangement has greatly improved.

The inspection of the C group on the 30th day post-operation confirmed a distinguished improvement in the re-epithelialization process, reforming the surface epithelium above the wound gap. At the same time, inflammatory signs remained noticeable (Figures 10A,C,E,G). Tracing mucosal healing in the AgNPs group at the same age demonstrated a distinct amelioration, with the regenerated epithelium overtaking the anastomotic area, restoring the typical architecture of the surface epithelium. The lamina propria was less edematous and had fewer suture material remnants (Figure 10B). The architecture of the crypts was nearly standard, with minimal evidence of an inflammatory response surrounding them (Figure 10D). The arrangement of smooth muscle cells in the lamina muscularis mucosae and tunica muscularis was significantly improved (Figure 10F). The connective tissue layers exhibited pronounced fibrocyte proliferation and an abundance of interconnecting collagen bundles (Figure 10H).

**Figure 11:**
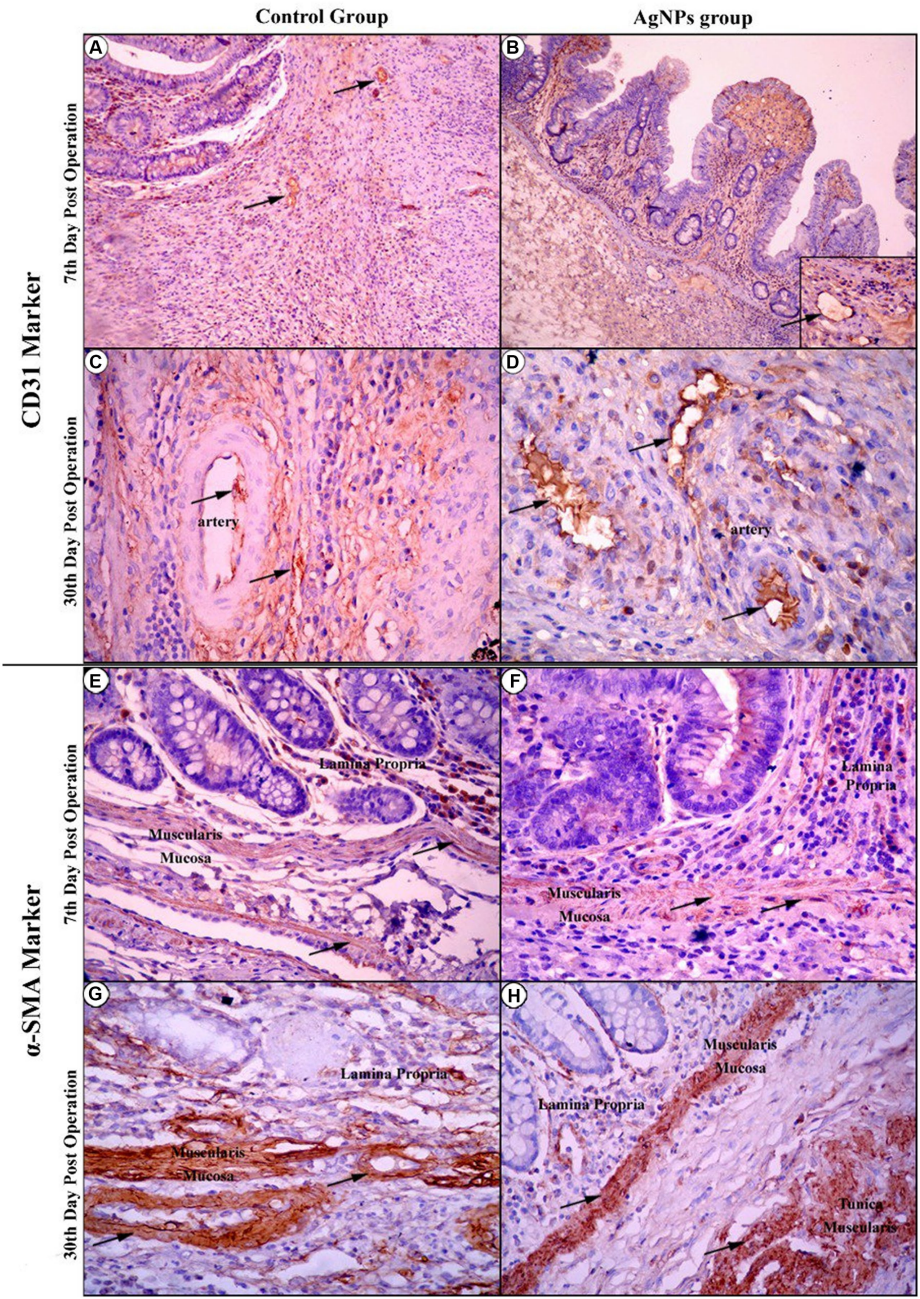
Immunohistochemical imaging of the CD31 marker at the 7th-day post-operation in the control and AgNPs groups revealed that endothelial cells of the four tunics of the cecum positively reacted to this marker (Arrows) **(A,B)**. On the 30th day post-operation, the control group had a greater increase in CD31-affiliated arterial and capillary vessels than the AgNPs group **(C,D)**. During Immunohistochemical imaging of the α-SMA marker at the 7th and 30th days post-operation, the control group expressed a lower affiliation of the α-SMA marker to smooth muscle fibers of mucosal layers **(E–G)** than appeared in the AgNPs groups **(F–H).**

The immunohistochemical imaging of the CD31 marker for both the C and AgNPs groups on the 7th day showed no significant difference (*p* > 0.05) in their microvessel count scores (35.75 ± 4.75 versus 34.13 ± 4.72, respectively), with the endothelial cells of the cecum’s four tunics positively reacting to this marker (Figures 11A,B). On the 15th day, the imaging showed increased CD31-affiliated microvessels in both groups within the immature granulation tissue. Nevertheless, a significant difference (*p* ≤ 0.05) was noted between them; the C group scored 50.88 ± 1.36, while the AgNPs group only scored 43.75 ± 1.04. At the end of the study (30th day post-operation), the C group maintained its progression, confirming the significant difference with a score of 67.50 ± 3.97, compared to the AgNPs group, which only scored 54.00 ± 3.46 (Figures 11C,D). The α-SMA expression intensity in myofibroblasts was compared to that of vascular smooth muscle cells. Despite the diverse intensities of staining, more than 90% of total cells were α-SMA positive. Therefore, tissues with α-SMA intensity lower than that of vascular smooth muscle cells were classified as having common expression, whereas those with α-SMA intensity higher than that of vascular smooth muscle cells were classified as having high expression. Throughout the trial, a positive correlation between both groups was maintained; in the C group, a low expression of α-SMA was recorded (Figures 11E,G), while the AgNPs group recorded a high expression of α-SMA (Figures 11F,H). Consequently, a statistically significant difference (*p* ≤ 0.05) in α-SMA expression between both groups was confirmed.

## 4 Discussion

Gastrointestinal stasis and obstructive disorders are the most common conditions observed in pet rabbits. They are considered life-threatening, so enterotomy is not recommended in intraluminal intestinal obstructions. In contrast, surgical management of the cecum or stomach is recommended for removing foreign objects; thus, the cecostomy model was chosen for this experiment (1). Therefore, a cecum transection and anastomosis surgery were selected as the model for the study. Various dressing materials fabricated with AgNPs are available for wound dressing, including AgNPs as nanocomposite materials, AgNPs as nanofibers, AgNPs as hydrogels, and AgNPs in semi-permeable film dressings. AgNPs as hydrogels can absorb exudates and maintain moisture in the wound environment promoting proper and rapid healing. Recently, AgNPs in semi-permeable film dressings, such as alginate, have shown excellent hydration properties, and a prolonged release of silver nanoparticles, making them potential wound dressings (36).

Silver has a high safety margin. For example, the toxic dose of silver is the oral reference dose (RfD) = 5 × 10^−3^ mg/kg-day in humans and 25–500 mg/kg/day in rats (37). Its toxicity is known as argyria, which causes a permanent blue-grayish pigmentation of the mucous membranes, eyes, and skin (38). Additionally, a previous study (39) has shown that the large size of nanoparticles (15 or 40 nm) has low penetration into the bloodstream, resulting in decreased toxicity incidences. Therefore, in the present study, the large size of AgNPs (15 nm) was chosen.

Intestinal anastomosis healing may be impaired by various systemic, local, and technical factors, specifically the development of early postoperative anastomotic strength. Restoring tissue strength is a crucial aspect of wound healing. This process necessitates adequate deposition of extracellular matrix components, specifically collagen fibers in the wound area (40). AgNPs have been extensively utilized as medical dressings for treating wounds and burns due to their antibacterial and anti-inflammatory properties compared to other silver agents (14). Moreover, AgNPs have been shown to more effectively inhibit inflammatory cell infiltration and reduce proinflammatory cytokines (8, 13, 41). The factors mentioned earlier may explain the causes of the decrease and narrowing of the diameter of the ultrasound scanning cecum at the anastomotic line in the C group compared to in the AgNPs group postoperatively.

In normal tissue restoration and healing following trauma or surgery, healing is preceded by various biological events, including exudation, granulation tissue formation, coagulation, and inflammation. Hence, inflammation plays an integral part in all these processes. The rapid inhibition of inflammatory cells and downregulation of proinflammatory cytokines are critical factors in tissue repair and subsequent remodeling for most wounds (42). In addition, applying multiple anti-inflammatory agents is considered an effective method for promoting tissue repair and regeneration.

However, a persistent inflammatory response can hinder wound healing due to inflammatory cell infiltration as well as proinflammatory cytokine upregulation. Therefore, anti-inflammatory agents, such as AgNPs, were utilized to accelerate healing (41). Therefore, this research aimed to determine the impact of silver alginate nanoparticles on the healing of rabbit cecum anastomosis.

Most patients developed anastomotic leakage within the first 2 weeks following surgery (10, 43), so rabbits were followed up 1 month following surgery in the current study. No mortality or significant complications have been reported. In addition, no clinical changes that would have suggested the development of ileus or sepsis were detected. The animals could maintain their weight; therefore, it was presumed that the material did not result in postoperative GI obstruction.

The anastomosis line in the current investigation was well-healed, as determined by macroscopic examination. Furthermore, there were no morphological alterations in the adjacent tissues or the entire abdominal cavity in the AgNPs groups; thus, the material appears safe to use. The degradation speed remains unanswered as the material used was still present in the place of application at the end of the experiment. This finding suggests that the material is not rapidly absorbed and does not tend to be readily assimilated. These results agreed with Liu et al. (9), which concluded that silver nanoparticle-coated sutures had beneficial positive effects on intestinal anastomosis in mice.

The incidence of postoperative adhesions in the peritoneal cavity was significantly reduced in the AgNPs group compared to the C group. The AgNPs group was introduced into fibrous membranes, and an *in vitro* investigation demonstrated that this novel membrane inhibited fibroblast proliferation and adhesions in addition to exhibiting antibacterial properties, as described by Liu et al. (10). Bursting pressure measurements aim to characterize the anastomosis’s stability functionally. The measures can provide insight into the completeness and stability of the healing process. Fluid instillation can reveal anastomoses prone to leaking or inadequate healing, primarily reflecting the mechanical integrity of the sutures, rather than the healing process (44). In this study, the rupture pressure of the anastomotic sites was substantially higher in the silver group than in the C group, indicating superior anastomotic area healing at each postoperative time point in the AgNPs group compared to the C group. Delayed or insufficient healing at the site of a bowel anastomosis can lead to anastomotic leakage with associated morbidity (45).

In our study, all animals in the AgNPs group (100%) showed no signs of anastomotic leakage, scoring one and revealing a better effect of AgNPs on wound healing at the anastomotic site, in contrast to the three animals in the C group, which showed leakage. Additionally, the degree of stenosis was significantly lower in the AgNPs group. These results may be attributed to AgNPs effectively reducing the infiltration of inflammatory cells, inhibiting the production of inflammatory cytokines, and upregulating the expression of matrix metalloproteinases (46).

With increasing postoperative days, the tensile strength of the anastomotic stoma substantially improved in both groups, reflecting an increased accumulation of collagen found on the 7th and 15th days postoperatively. These findings are consistent with those of Tang et al. (33), which investigated the influence of anastomotic fistula recovery time in rabbits.

Numerous cells and communication pathways are involved in the cellular level process of intestinal self-healing after injury. During this process, epithelial cells migrate and proliferate, immune cells are activated, and growth factors and cytokines are produced, thus restoring normal intestinal mucosal function and structure (47).

However, disruptions in this process can lead to chronic inflammation and diseases such as inflammatory bowel disease. Owing to their ability to promote tissue healing, AgNPs have piqued the interest of researchers as a viable option for medical dressings (40). This is due to their potent antibacterial, antioxidant, anti-inflammatory, and wound-healing properties. Therefore, in this study, the ability of AgNPs to accelerate the anastomotic lesion repair of the cecum was evaluated (11).

In the early stages of the study, histological examination of the AgNPs-treated group revealed bundles of fibrin threads embedded in diminishing necrotic debris, indicative of an accelerated clotting cascade. Conversely, the C group did not exhibit these signs, which corroborated previous studies (48, 49) on the procoagulant activity of AgNPs.

Moreover, tunics with mild cellular hyperplasia of the crypt showed less impact on inflammation around the wound edges, a promising early sign of epithelial restitution. This is consistent with Fraser et al. (50), which demonstrated that AgNPs successfully mitigate the inflammatory effect, decreasing inflammatory cell infiltration, blocking proinflammatory cytokines, reducing lymphocyte and mast cell infiltration, and boosting wound healing efficacy by enhancing neutrophil apoptosis.

A recent study (8) also found that AgNPs improved wound healing and cosmetic appearance in an animal model. In the mid-stages of the study, debris and pathogens were removed, and inflammatory cells prepared the wound for the next phase of cell proliferation, during which fibroblasts and epithelial cells were recruited and activated.

During the subsequent healing phase, the presence of histiocytes revealed that the AgNPs group exhibited a remarkable increase in the proliferation and regeneration rates of the entire intestinal wall (9, 11). This progress can largely be attributed to the elevated angiogenesis rates.

In the subsequent age analysis, despite the importance of neo-angiogenesis in advancing anastomotic healing by increasing oxygen supply and nutrient delivery to the wound bed (16), the AgNPs group displayed a lower microvessel count. This finding indicates a low number of leukocytic cells, suggesting that the wound was in its advanced stage of healing and did not require higher angiogenesis rates. Similarly, the typical architecture of the mucosal surface was restored, likely caused by the complete overrun of the anastomotic area by the migrated progeny of crypt cells on the surface epithelium (11).

The presence of acute crypt enlargement, moderate hyperplasia, and elevated mitotic figures at the base of the crypts primarily supported this restoration (21). Macroscopic examination of the cecal wall revealed no alterations or hemorrhage at the anastomotic site, confirming these observations. As anastomotic healing progressed, there was a significant increase in the number of initial fiber-producing cells, forming a collagen matrix from immature cells. This thick collagen deposition was then reorganized and remodeled into tight collagen bundles, providing the anastomotic tissue with stability. Compared to the C group, the high intensity of α-SMA expression in the AGNPs group suggested the widespread presence of myofibroblast cells, which are responsible for producing the contractile force that causes the wound region to contract throughout the healing process. This observation was previously supported by You et al. (51).

Overall, the data presented in this study supported the idea that a silver nanoparticle sheet could reduce inflammation, stimulate neovascularization, and enhance intestinal wound healing in a rabbit model of cecal anastomosis. These results may serve as a theoretical basis for the clinical prevention of intestinal anastomosis.

## Data availability statement

The original contributions presented in the study are included in the article/supplementary material, further inquiries can be directed to the corresponding author.

## Ethics statement

The animal studies were approved by the Mansoura University Animal Care and Use Committee with a documented code MU-ACUC (MV.R.20.03.110). The studies were conducted in accordance with the local legislation and institutional requirements. Written informed consent was obtained from the owners for the participation of their animals in this study.

## Author contributions

ZA: Investigation, Methodology, Writing – original draft. RF: Data curation, Investigation, Resources, Writing – original draft. AF: Conceptualization, Methodology, Writing – review & editing. AMA: Investigation, Resources, Writing – original draft. WM: Investigation, Methodology, Resources, Writing – original draft. AAA: Conceptualization, Methodology, Writing – review & editing, Project administration, Resources. MA: Conceptualization, Formal analysis, Investigation, Methodology, Supervision, Visualization, Writing – original draft, Writing – review & editing.
